# Effect of the Web-Based Intervention MyPlan 1.0 on Self-Reported Fruit and Vegetable Intake in Adults Who Visit General Practice: A Quasi-Experimental Trial

**DOI:** 10.2196/jmir.5252

**Published:** 2016-02-29

**Authors:** Jolien Plaete, Geert Crombez, Celien Van der Mispel, Maite Verloigne, Vicky Van Stappen, Ilse De Bourdeaudhuij

**Affiliations:** ^1^ Ghent University Ghent Belgium

**Keywords:** eHealth, Web-based intervention, dietary interventions, fruit and vegetable intake, general practice, self-regulation, health promotion, primary prevention

## Abstract

**Background:**

Web-based interventions typically have small intervention effects on adults’ health behavior because they primarily target processes leading to an intention to change leaving individuals in an intention-behavior gap, they often occur without contact with health care providers, and a limited amount of feedback is provided only at the beginning of these interventions, but not further on in the behavior change process. Therefore, we developed a Web-based intervention (“MyPlan 1.0”) to promote healthy behavior in adults. The intervention was based on a self-regulation perspective that also targets postintentional processes and guides individuals during all phases of behavior change.

**Objective:**

The study investigated the effectiveness of MyPlan1.0 on fruit and vegetable intake of Flemish adults visiting general practice (3 groups: control group, intervention group recruited by researchers, and intervention group recruited and guided by general practitioners [GPs]). Second, it examined whether there was a larger intervention effect for the intervention group guided by GPs compared to the intervention group recruited by researchers.

**Methods:**

Adults (≥18 years) were recruited in 19 Flemish general practices. In each general practice, patients were systematically allocated by a researcher either for the intervention group (researchers’ intervention group) or the waiting-list control group that received general advice. In a third group, the GP recruited adults for the intervention (GPs intervention group). The two intervention groups filled in evaluation questionnaires and received MyPlan 1.0 for a behavior of choice (fruit, vegetable, or physical activity). The waiting-list control group filled in the evaluation questionnaires and received only general information. Self-reported fruit and vegetable intake were assessed at baseline (T0), 1 week (T1), and 1 month (T2) postbaseline. Three-level (general practice, adults, time) linear regression models were conducted in MLwiN.

**Results:**

A total of 426 adults initially agreed to participate (control group: n=149; GPs’ intervention group: n=41; researchers’ intervention group: n=236). A high attrition rate was observed in both intervention groups (71.8%, 199/277) and in the control group (59.1%, 88/149). In comparison to no change in the control group, both the GPs’ intervention group (fruit: χ^2^_1_=10.9, *P*=.004; vegetable: χ^2^_1_=5.3, *P*=.02) and the researchers’ intervention group (fruit: χ^2^_1_=18.0, *P*=.001; vegetable: χ^2^_1_=12.8, *P*<.001) increased their intake of fruit and vegetables.

**Conclusions:**

A greater increase in fruit and vegetable intake was found when the Web-based intervention MyPlan 1.0 was used compared to usual care of health promotion in general practice (ie, flyers with general information). However, further investigation on which (or combinations of which) behavior change techniques are effective, how to increase response rates, and the influence of delivery mode in routine practice is required.

**Trial Registration:**

ClinicalTrials.gov NCT02211040; https://clinicaltrials.gov/ct2/show/NCT02211040 (Archived by WebCite® at http://www.webcitation.org/6f8yxTRii)

##  Introduction

A healthy diet, more specifically a diet rich in fruit and vegetables, can prevent chronic diseases (eg, hypertension, coronary heart disease, diabetes) in adults [1]. Therefore, the World Health Organization recommends adults to consume at least 5 portions or 400 g of fruit and vegetables per day [2]. However, 78% of the adult population worldwide consumes less than 5 portions of vegetables and fruit daily [3]. Western adults (in Belgium, Luxembourg, France, Ireland, The Netherlands, Great Britain) consume, on average, only 129 g of fruit and vegetables per day [1]. In Belgium, adults are recommended to consume 3 portions of fruit and 300 g of vegetables per day. However, only 38% and 47% of Belgian adults fulfill these norms for fruit and vegetable intake [4]. Thus, an effective intervention to promote fruit and vegetable intake is needed.

Web-based interventions are promising to change dietary behavior and allow a personalized approach at a relatively low cost by making use of interactive, computerized technologies [5,6]. They provide several advantages, such as reduced personal demands, consistency over time, increased interactivity, flexibility, automated data collection, and more honest self-reporting. However, the effects of previously conducted Web-based interventions on adults’ health behavior are generally small [7,8]. This may be because Web-based interventions target primarily variables that address the adoption of an intention to change (eg, knowledge), hence leaving many individuals in an intention-behavior gap. Interventions based on social cognitive theories primarily address determinants that influence individuals’ intention to change. However, intentions do not automatically translate into behavior because of competing demands and unforeseen obstacles. Therefore, to overcome this so-called intention-behavior gap, postintentional factors, such as self-regulation skills and strategic planning, are needed [9]. Self-regulation refers to internal and/or transactional processes that enable individuals to guide their goal-directed activities to translate their intentions into behavior over time and across changing circumstances or contexts [10]. Therefore, a self-regulation perspective can be used to target both pre- and postintentional processes, and to develop interventions that guide individuals during all phases of behavior change [11-13]. The systematic review of Greaves et al [14] showed that increased effectiveness of dietary and physical activity interventions was also associated with using self-regulation behavior change techniques, increased contact frequency, engaging social support, targeting both diet and physical activity, and using well-defined behavior change techniques. The meta-analysis of Michie et al [15] also provides evidence to include behavior change techniques that target both preintentional and postintentional processes, namely prompting intention formation and goal setting, providing feedback on performance, prompting review of goal progress, and self-monitoring. Finally, another meta-analysis of Lara et al [16] showed that barrier identification/problem solving, plan social support, use of follow-up prompts, and goal setting were effective in increasing fruit and vegetable intake.

Another possible reason for the small effects of Web-based interventions may be the limited amount of feedback that is provided at the very beginning of these interventions, but not further on in the process of behavior change. To target this problem, it is recommended to include personal feedback at several moments. This means that participants receive feedback during the actual process of behavioral change [7,14].

A pertinent problem of Web-based interventions is the low percentage of individuals who start with an Internet-delivered intervention and low sustained use of Internet-delivered interventions [17]. Most existing computer-tailored interventions are self-guided without direct contact with an expert or therapist (8). However, enhanced use and larger effects were found in Web-based intervention studies in which personal contact was included [17-20]. Delivery of Web-based interventions in general practice may have advantages. General practitioners (GPs) already play a role in the promotion of healthy nutrition in adults, patients trust their GP as a reliable source of information concerning nutrition [21,22], and Web-based interventions may take over some tasks of the GPs. Web-based interventions can also prompt and guide GPs to further counsel their patients. Furthermore, direct contact with a GP may result in more tenacious goal engagement of the patients and, thus, a more effective intervention [23]. However, several barriers to incorporating health promotion interventions in general practice were also reported: lack of training and skills, lack of time, patient reluctance, other priorities in patient care, and lack of resources [23].

Based on these findings and suggestions, we developed a new Web-based intervention (“MyPlan 1.0”) to promote healthy behavior in adults [24]. The intervention was based on self-regulation theory. Barriers to implement the intervention through general practice were taken into account by using a Web-based program and involving GPs in the development process [23,24]. Behavior change techniques that were incorporated were tailored feedback, barrier identification, problem solving, goal setting, implementation intentions, follow-up session with goal evaluation, stimulating social support, and prompting self-monitoring. Preintentional processes were targeted with personal feedback through which awareness was raised [9,13]. Postintentional processes were addressed with action planning, problem solving, prompting to self-monitor behavior, and follow-up modules that provided repeated feedback and guidance based on whether and how individuals changed their behavior and reached their goals. To deliver the Web-based intervention in general practice, tablet computers and flyers were used.

The aim of this study was to test the effectiveness of the Web-based intervention on fruit and vegetable intake in Flemish adults visiting general practice (3 groups: control group, intervention group recruited by researchers in general practice, and intervention group recruited and guided by GPs). A second study aim was to specifically examine whether there was a larger intervention effect on fruit and vegetable intake for the intervention group recruited by GPs compared to the intervention group recruited by researchers.

## Methods

### Study Design and Participants

A cluster quasi-experimental trial was used to evaluate the effects of the self-regulation Web-based intervention delivered through general practice on adults’ fruit and vegetable intake. Potential participants were recruited in general practices in Flanders (Northern part of Belgium). A convenience sample of general practices was recruited by using email messages, telephone calls, and advertisements on association websites of GPs. In total, 19 general practices, of which 6 solo practices (only 1 GP) and 13 group practices (more than 1 GP), agreed to participate in the study. In each general practice, three groups were recruited. In each practice, researchers systematically allocated at least 10 participants to an intervention group (researchers’ intervention group, n=190 adults) and at least 10 participants to a waiting-list control group (n=190 adults). That is, alternating between morning and evening consultations, participants were either invited to participate in the intervention group or in the control group. In each practice, the GP also recruited adults for the intervention (GPs’ intervention group). Each GP was instructed to recruit at least 10 adults visiting their practice who were age 18 years or older (n=190 adults). Various options of delivering MyPlan 1.0 to the patients were provided, and GPs selected the one that they considered appropriate for the situation or patient. The options were illustrated to GPs using a flowchart (see Multimedia Appendix 1). During the study period, GPs received weekly telephone-call reminders to recruit patients and to evaluate the study procedure.

Both intervention groups filled in evaluation questionnaires on health behavior and received the self-regulation Web-based intervention MyPlan 1.0, which will be further explained in Methods. The waiting-list control group also filled in the evaluation questionnaires on health behavior and received general information about health behavior (general recommendations on fruit and vegetable intake and advantages of achieving the recommendations for fruit and vegetable intake).

Only Dutch-speaking adults who were 18 years or older and had access to the Internet were eligible. Interested and eligible adults could enroll by filling out an informed consent and a short questionnaire in which general information (name, email address, telephone number, and GP’s name) was gathered. Data were collected from November 2014 to June 2015. The study protocol was approved by the Ghent University Ethics Committee. The trial protocol of this study is reported at ClinicalTrials.gov (ClinicalTrials.gov: NCT02211040).

### MyPlan 1.0: Fruit and Vegetable Components

MyPlan 1.0 is a Web-based intervention [25] developed using self-regulation theory [9,13], and the Health Action Process Approach model [9]. It focuses on two different behaviors: healthy eating (ie, fruit and vegetable intake) and physical activity. Therefore, different intervention modules were developed, including a fruit and vegetable module, the effects of which are reported in this paper.

The intervention content is described in more detail in the study protocol paper of MyPlan 1.0 [24]. In the first module (T0), both preintentional processes that lead to a behavioral intention and postintentional processes that lead to actual behavioral change were addressed. Preintentional processes were addressed by generating personal feedback to raise awareness and to motivate adults to change their behavior. Individuals filled in a questionnaire on a particular health behavior. Based on their answers, personal feedback was provided. The personal level of the health behavior was discussed and compared to health guidelines. Adults were provided with the possibility to read more information about the behavior (eg, relation with diseases and health, benefits). This is akin to previous computer-tailored programs; hence, the content was largely based on previously developed interventions [26,27].

Postintentional processes were addressed by facilitating action planning. Participants were invited to make an action plan to bridge the gap between intentions and behavior. Adults were guided through action planning by answering questions in the tool. Participants were asked how many portions of fruits/vegetables they wanted to eat (eg, eating 2 portions of fruit), on how many days (eg, every day), when (eg, during breakfast and as a snack during the afternoon), and where (eg, at home). Adults were also offered the possibility to identify difficult situations and hindering factors (ie, coping planning). This was achieved by selecting relevant options from a predefined list of hindering factors and barriers. Based on these selections, several solutions were listed and participants could select the solutions that they considered relevant for their situation and wanted to apply. Adults were guided to make an if-then plan (eg, if I’m hungry in the afternoon, then I eat an apple instead of a candy bar). Adults were also advised on how to self-monitor their behavior (eg, using an agenda) and to pursue their health goals as stated in their action plan. The personal action plan was sent via email and adults were offered the possibility to send the action plan to family or friends for social support.

Adults in both intervention groups were informed that they had the opportunity to discuss their feedback or action plan with their GP during their following consultation. That way, patients’ personal advice could be used by GPs to talk about patients’ health behavior and to discuss attainability of patients’ goals. Module 2 (T1) was activated 1 week after finishing module 1. Participants were contacted by email to revisit the website to complete module 2. In this follow-up module, adults received repeated feedback about their behavior change process (eg, ate more or less pieces of fruit compared to last week) and their goals (eg, did or did not reach the goal to eat 3 pieces of fruit every day). Thereafter, adults had the possibility to adapt their action plan. Adaptations could consist of formulating new goals (eg, more feasible, a further goal if the identified goal was reached), or of reconsidering coping plans based on the experienced difficulties and barriers during goal pursuit. Module 3 (T2) was activated 1 month after finishing module 1, and was identical to module 2. We investigated the change in fruit and vegetable intake from T0 to T2.

### Procedure

**Figure 1:**
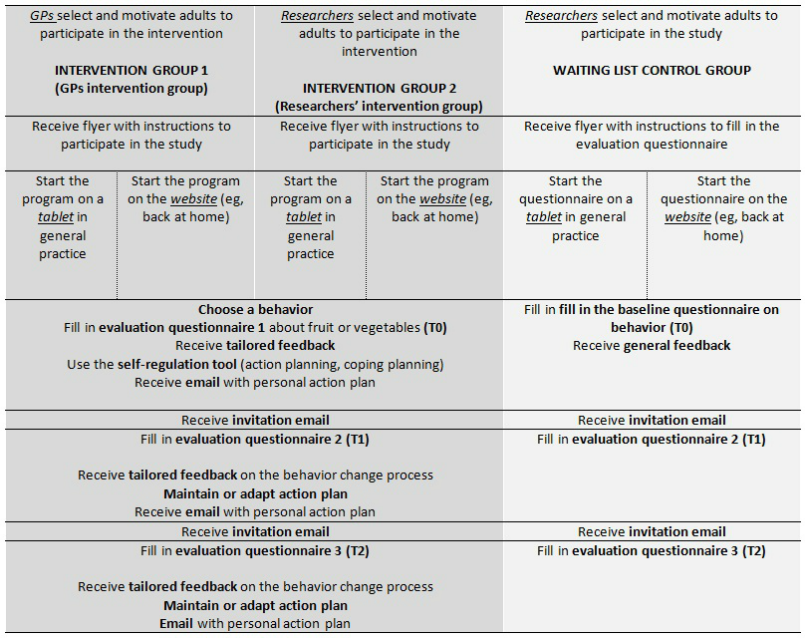
Study procedure.

In general practice, adults were either assigned to the intervention group or to the control group (2:1). Therefore, adults received a flyer with a personal code that gave access to the Web-based program (intervention groups) or questionnaires only (control group) (Figure 1).

Adults in the intervention condition could choose to log in to the computer-tailored program website on a tablet available in at the general practice or take a flyer with a referring link on it and log in to the website elsewhere (eg, on their computer, when back at home or at their workplace). After logging in to the website, adults in the intervention group could choose a behavior of their interest (ie, fruit, vegetables, or physical activity). After participants chose a behavior, they received access to the intervention component of the chosen behavior, filled in the baseline questionnaire on the chosen behavior (T0), and ran the first session of the chosen health behavior. For this study, only adults that chose to focus on fruit and/or vegetable intake and who completed these behavior components of the intervention were included.

Participants in the waiting-list control group were asked to fill in the baseline questionnaire on behavior (T0). After they filled in the questionnaire, they received general feedback on the website. Adults in the control group logged in to the website and filled out the online questionnaire, similar to the participants of the intervention groups. However, they only received nontailored, general information regarding health norms as feedback.

Adults who started on a tablet at the general practice and who were not able to complete the first session (eg, not enough time in the waiting room) could halt the program and resume it at any time by logging in again on the website. Participants who did not start or complete the first session after 1 week received a reminder email.

### Measurements

Demographic variables were assessed in the online questionnaire at baseline (T0) and included age, sex, height, weight, and highest degree of education (primary or secondary education, college, university). Fruit intake was measured via the “fruit test.” The reported pieces of fruit per week were multiplied with the correct portion size of the corresponding types of fruit to calculate the mean grams per week. To calculate the mean portion size of fruit per day, mean grams of fruits per week were divided by 7 and 125 (one portion of fruit is equal to 125 g). For participants who selected vegetable intake as the intervention target behavior, the mean grams of vegetables per day were calculated by using the “vegetable test.” The reported portions of vegetables were multiplied with the correct portion size of the corresponding vegetable and divided by 7 to calculate the mean grams per day. In both the fruit test and vegetable test, participants reported how many days in the past 7 days they ate fruit/vegetables. If participants ate fruit/vegetables, a list with fruits/vegetables that are frequently eaten in a Western diet was displayed on the screen. For each type of fruit or vegetable, portion sizes and household sizes were described (eg, 1 cherry=4 g, a dessert plate of berries weighs approximately 100 g). Participants were instructed to indicate for each type of fruit or vegetable the number of portions they ate during the past 7 days. The reported portions of fruit/vegetables were multiplied with the portion size of the corresponding types of fruit/vegetables and divided by 7 to calculate the mean grams per day. Criterion validity, assessed against a 7-day diary, was substantial for the fruit test, with a Spearman rho value of .73 and moderate for the vegetable test with a Spearman rho value of .52.

### Statistical Analysis

To check normality of the dependent variables (mean portion of fruit and mean portion of vegetables), a Kolmogorov-Smirnov test in SPSS Statistics version 22.0 (SPSS Inc, Chicago, IL, USA) was conducted and showed that the data were not normally distributed. To correct for positive skewness, the mean portion of fruits and mean portion of vegetables were log-transformed. For ease of interpretation, back-transformed mean values are reported in the tables.

Independent sample *t* tests (for quantitative data) and chi-square tests (for qualitative data) in SPSS were used to compare participants’ characteristics at baseline between both intervention groups and the control group and to conduct dropout analyses. Little missing completely at random (MCAR) tests were conducted to test whether the missing values were completely at random.

Due to the hierarchical structure of the data, with 496 adults being nested within 19 general practices, we conducted multilevel analyses with three levels (general practice, adults, and time) to investigate the intervention effect on fruit and vegetable intake from T0 to T2. The iterative generalized least squares (IGLS) estimation method in MLwiN (version 2.32) was used to conduct the multilevel regression analyses. Completer analyses were conducted first, followed by intention-to-treat analyses in which missing values at T2 were replaced by mean fruit intake values from T0 or T1 (assuming that patients lost to follow-up at T2 did not change their behavior reported at T0 or T1).

First, a 3-level null model (general practice, adults, time) including the dependent variable only was estimated for fruit intake (null model 1) and vegetable intake (null model 2). The null models were used to show the percentage of the total variance by changes in time (level 1), differences among adults (level 2), and differences among GPs (level 3).

Second, age, gender, educational level, and body mass index (BMI) were inserted in the models as covariates for fruit intake (model 1a) and in the model for vegetable intake (model 2a). Likelihood ratio tests were conducted to compare both models with their respective null model. The model with covariates was considered to have a better fit than the null model, if the likelihood ratio test was statistically significant.

Third, time and condition were included as predictors in both models (model 1b, model 2b). In these models, we also investigated whether changes in fruit intake and vegetable intake over time (before and 1 month after the intervention) differed for adults in the three conditions by exploring the interaction effect between time and condition (time × condition). Likelihood ratio tests were used to determine if the models with predictors were better fits than the models with only covariates.

In the Results section, we first report the null model, model a, and model b conducted with completer analyses; second, we report model b again conducted with intention-to-treat analyses. Statistical significance was set at a level of .05.

## Results

### Participant Characteristics, Response, and Dropout Analysis

**Figure 2:**
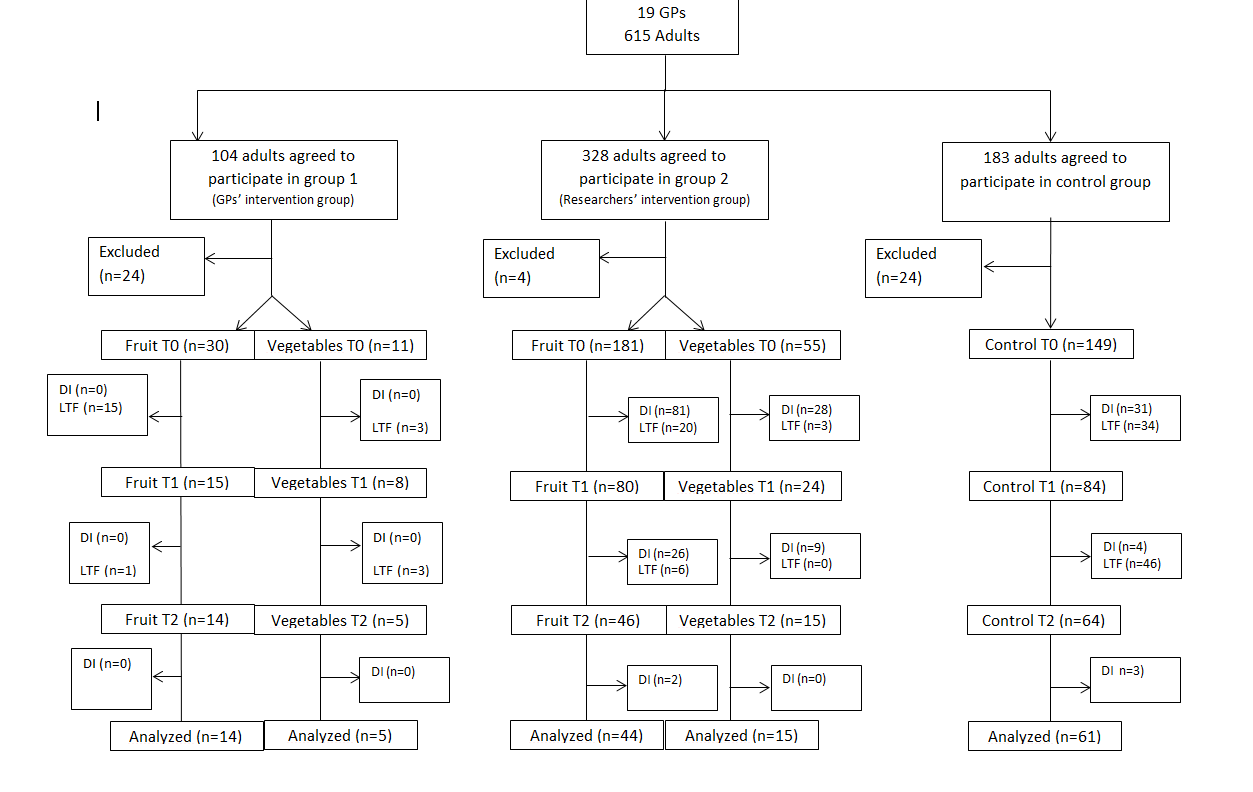
Participants' flow through study. DI: discontinued intervention; LTF: lost to follow-up.

Figure 2 shows the flow of participants. In total, 615 adults agreed to participate by signing the informed consent. Of these participants, 104 adults were in intervention group 1 (GPs’ intervention group), 328 in intervention group 2 (researchers’ intervention group), and 183 in the control group at baseline (T0). In the intervention groups, fruit intake was initially selected by 211 participants (30 in GPs’ intervention group, 181 in researchers’ intervention group) and vegetable intake was initially chosen by 66 participants (11 in GPs’ intervention group, 55 in researchers’ intervention group).

Dropout analyses (at T2) indicated that men (χ^2^_1_=15.9, *P*<.001), participants with low education (χ^2^_1_=11.9, *P*<.001), and participants who chose more than one behavior (χ^2^_1_=6.1, *P*=.01) were more likely to drop out. No significant differences were found for condition, age, fruit/vegetable intake at baseline, meeting health guidelines, and BMI. A Little MCAR test showed that missing values were completely at random for fruit intake (χ^2^_2_=1.7, *P*=.42) and for vegetable intake (χ^2^_8_=12.4, *P*=.13).

Baseline characteristics are shown in Table 1. Participants in the researchers’ intervention group had a significantly lower fruit intake compared to those in the GPs’ intervention group (*t*_98_=–3.08, *P*=.002) and compared to those in the control group (*t*_433_=2.93, *P*=.004). Also, participants’ BMI was different between the control group and the GPs’ intervention group for fruit intake, with adults in the GPs’ intervention group having a higher BMI, but these results were not statistically significant (*t*_286_=1.72, *P*=.09). Furthermore, more participants in the GPs’ intervention group for vegetable intake had a higher education than the researchers’ intervention group for vegetable intake (χ^2^_1_=4.0, *P*=.04).

**Table 1:** Baseline characteristics of participants (N=314).

Characteristics		Fruit intake		Vegetable intake		Control group (n=118)
		GPs’ intervention (n=30)	Researchers’ intervention (n=100)	GPs’ intervention (n=11)	Researchers’ intervention (n=55)	
Age (years), mean (SD)		43.68 (11.38)	44.20 (13.74)	45.80 (14.95)	43.53 (13.59)	46.14 (14.76)
Gender (male), n (%)		8/30 (27)	31/100 (31.0)	4/11 (36)	36/55 (65)	39/118 (33.1)
Education level (high university or college), n (%)		17/30 (57)	48/100 (48.0)	7/11 (64)^a^	19/55 (35)^a^	58/118 (49.2)
BMI (kg/m^2^), mean (SD)		26.50 (5.11)^b^	25.99 (5.65)	25.64 (6.12)	26.51 (6.38)	25.21 (5.10)^b^
Not meeting recommendations, n (%)						
	Fruit	28/30 (93)	94/100 (94.0)	—	—	106/118 (89.8)
	Vegetables	—	—	10/11 (91)	50/55 (91)	110/118 (93.2)
Fruit intake (portion/day), mean (SD)		1.60 (26.50)^a^	1.13 (1.01)^a,c^	—	—	1.44 (1.20)^c^
Vegetable intake (g/day), mean (SD)		—	—	120.00 (107.34)	153.19 (115.79)	141.63 (86.33)

^a^ Significant difference between GPs’ intervention group and researchers’ intervention group (*P*<.05).

^b^ Significant difference between GPs’ intervention group and control group (*P*<.05).

^c^ Significant difference between researchers’ intervention group and control group (*P*<.05).

### Reaching Health Guidelines

At T0, only a minority of the participants reached the health norm for fruit intake (7%, 2/30 in the GPs’ intervention group; 6.0%, 6/100 in the researchers’ intervention group; 10.1%, 12/118 in the control group), but no significant association between the different conditions was found (χ^2^_2_=2.7, *P*=.26). A significant association between the different conditions and reaching health guidelines for fruit intake at T2 was found (χ^2^_2_=18.4, *P*<.001). More adults in the intervention groups—57% (8/14) in the GPs’ intervention group and 20% (9/44) in the researchers’ intervention group—reached the health norm for fruit intake at T2 than adults in the control group (8%, 5/61). For vegetable intake, only a minority reached the health guidelines at T0 (9%, 1/11 in the GPs’ intervention group; 9%, 5/55 in the researchers’ intervention group; and 6.8%, 8/118 in the control group) and no significant association with condition was found (χ^2^_2_=0.6, *P*=.73). At T2, a significant association between the different conditions and reaching health guidelines for vegetable intake was reported (χ^2^_2_=18.5, *P*<.001). More adults in the intervention groups—20% (1/5) in the GPs’ intervention group and 60% (9/15) in the researchers’ intervention group—reached the health norm for vegetable intake at T2 than adults in the control group (8%, 5/61).

### Effects on Fruit Intake

In the completer analyses, the random parts of the null model for fruit intake showed that the variance at both the time level (61%, χ^2^_1_=61.8, *P*<.001) and adult level (39%, χ^2^_1_=19.2, *P*<.001) differed significantly from zero. There was no significant between-GP variance in adults’ fruit intake. Of the covariates that were included in the model, only age was significantly related to fruit intake. Higher age was related to higher fruit intake (β=0.011, SE 0.005; χ^2^_1_=4.4, *P*=.04) (see Table 2).

**Table 2:** Relationship with age, gender, educational level, BMI, time, condition and the interaction of time×condition with fruit intake.

Parameter		Null model 1	Model 1a	Model 1b
**Fixed part, β (SE)**				
	Intercept	1.491 (0.04)	1.494 (0.142)	0.199 (0.148)
	Age		0.012 (0.005)^a^	0.011 (0.005)^a^
	Gender		0.024 (0.155)	0.043 (0.153)
	Educational level		–0.093 (0.145)	–0.142 (0.143)
	BMI		–0.038 (0.151)	–0.012 (0.149)
	Condition			0.199 (0.148)
	Time			0.035 (0.113)
	Time×condition			–0.883 (0.268)^b^
**Random part, σ** ^**2**^ **(SE)**				
	Time-level variance	1.063 (0.135)^b^	0.570 (0.077)^b^	0.420 (0.057)^b^
	GP-level variance	0.000 (0.000)	0.010 (0.034)	0.000 (0.000)
	Adult-level variance	0.692 (0.158)^b^	0.722 (0.123)^b^	0.780 (0.110)^b^
Deviance test model		1211.12	995.81	956.231
χ^2^(*df*)			215.3 (4)^b^	254.9 (7)^b^

^a^ *P*<.05

^b^ *P*<.001.

**Figure 3:**
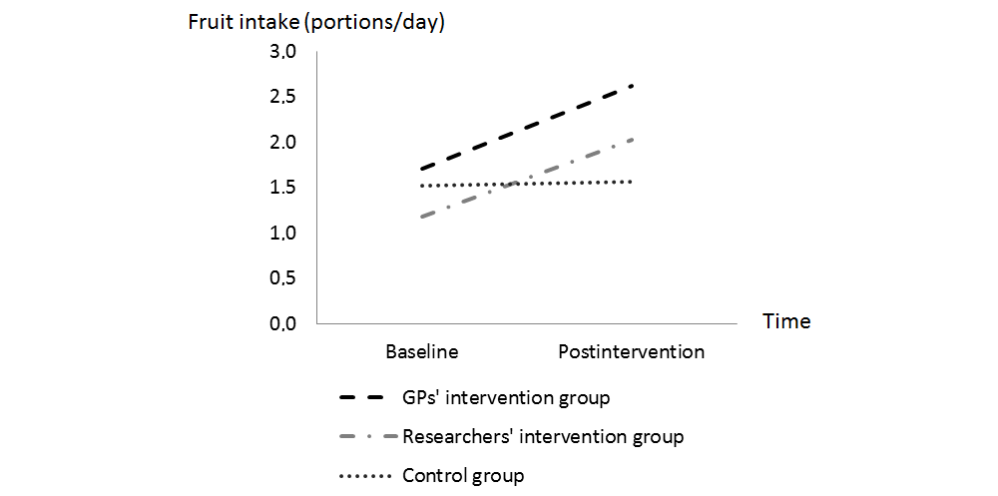
Changes in fruit intake from baseline (T0) to postintervention (T2) in the three different groups.

In the completer analyses, a significant interaction effect for fruit intake was found, suggesting that the change in fruit intake over time (from T0 to T2) significantly differed between the control group and the GPs’ intervention group (β=–0.883, SE 0.268; χ^2^_1_=10.9, *P*=.004) and between the control group and the researchers’ intervention group (β=–0.802, SE 0.189; χ^2^_1_=18.0, *P*=.001). Table 3 shows a greater increase in fruit intake from baseline to posttreatment in the researchers’ intervention group and in the GPs’ intervention group compared to in the control group (see also Figure 3). The change in fruit intake did not significantly differ between both intervention conditions (β=–0.081, SE 0.286; χ^2^_1_=0.1, *P*=.96).

**Table 3:** Change in fruit and vegetable intake from baseline (T0) to posttreatment (T2) for the three conditions.

Time		GPs’ intervention group	Researchers’ intervention group	Control group
**Fruit intake (portion/day), mean (SD)**				
	Baseline	1.70 (0.25)	1.18 (0.17)	1.52 (0.15)
	Posttreatment	2.62 (0.30)	2.02 (0.21)	1.56 (0.17)
**Vegetable intake (g/day), mean (SD)**				
	Baseline	115.00 (37.30)	145.43 (19.96)	118.09 (16.09)
	Posttreatment	266.88 (50.50)	291.09 (30.24)	143.02 (19.03)

The intention-to-treat analysis showed the same results: the change in fruit intake over time significantly differed between the control group and the GPs’ intervention group (β=0.403, SE 0.197; χ^2^_1_=4.2, *P*=.04) and between the control group and the researchers’ intervention group (β=0.328, SE 0.130; χ^2^_1_=6.4, *P*=.01). The change in fruit intake from baseline to postintervention did not significantly differ between both intervention conditions (β=0.075, SE 0.202; χ^2^_1_=0.1, *P*=.71).

### Effects on Vegetable Intake

In the completer analyses, the random parts of the null model for vegetable intake showed that the variance at both the time level (72.4%, χ^2^_1_=44.1, *P*<.001) and adult level (19.3%, χ^2^_1_=3.3, *P*=.07) differed significantly from zero. There was no significant between-GP variance in adults’ vegetable intake. A higher educational level was related to a higher vegetable intake (β=29.039, SE 16.269; χ^2^_1_=3.2, *P*=.07) and a higher BMI was related to a lower vegetable intake (β=–2.617, SE 1.471; χ^2^_1_=3.2, *P*=.08), but neither of these were statistically significant (see Table 4).

**Table 4:** Relationship with age, gender, educational level, BMI, time, condition, and the interaction of time×condition with vegetable intake.

Parameter		Null model 2	Model 2a	Model 2b
**Fixed part, β (SE)**				
	Intercept	160.964 (10.921)	140.072 (15.531)	118.094 (16.093)
	Age		0.472 (0.567)	0.437 (0.534)
	Gender		19.575 (16.771)	19.349 (15.484)
	Educational level		29.039 (16.269)	27.949 (15.484)
	BMI		–2.617(1.471)	–2.659 (1.375)
	Condition			27.340 (20.262)
	Time			24.931 (15.876)
	Time × Condition			120.734 (33.730)^a^
**Random part, β (SE)**				
	Time-level variance	10017.922 (1509.309)^a^	9861.971 (1519.984)^a^	8656.535 (1331.062)^a^
	GP-level variance	1144.326 (730.174)	1719.047 (920.754)	1388.825 (756.336)
	Adult-level variance	2671.313 (1473.540)	2252.752 (1454.147)	1700.278 (1245.686)
Deviance test model		3229.283	3075.562	3036.429
χ^2^ (*df*)			153.7 (4)^a^	192.9 (7)^a^

^a^ *P*<.001

**Figure 4:**
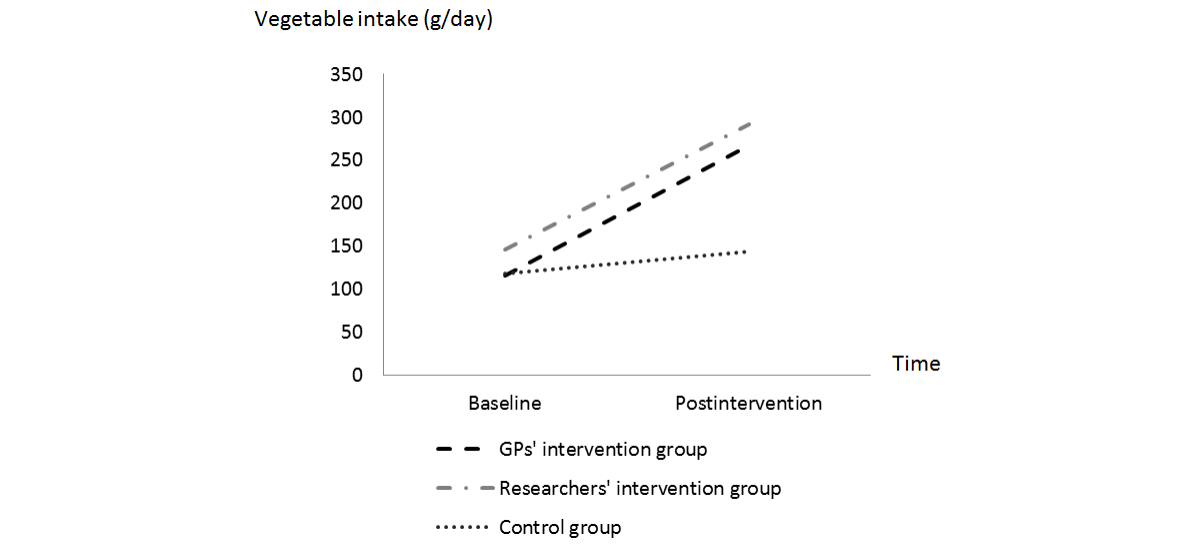
Changes in vegetable intake from baseline (T0) to postintervention (T2) in the three different groups.

In the completer analyses, there was a significant interaction effect for vegetable intake, suggesting that the change in vegetable intake over time (from T0 to T2) significantly differed between the control group and the GPs’ intervention group (β=126.944, SE 55.377; χ^2^_1_=5.3, *P*=.02) and between the control group and the researchers’ intervention group (β=120.734, SE 33.730; χ^2^_1_=12.8, *P*<.001). Table 3 shows a greater increase in vegetable intake from baseline to posttreatment in the GPs’ intervention group and in the researchers’ intervention group compared to in the control group (see also Figure 4).

Changes in vegetable intake from baseline to posttreatment did not significantly differ between both intervention conditions (β=–6.210, SE 60.874; χ^2^_1_=0.0, *P*=.92) in the completer analyses.

The intention-to-treat analysis also showed that changes in vegetable intake over time significantly differed between the control group and the GPs’ intervention group (β=93.020, SE 34.954; χ^2^_1_=7.1, *P*=.008) and a difference was found between the control group and the researchers’ intervention group but it was not statistically significant (β=29.648, SE 17.837; χ^2^_1_=2.8, *P*=.09).

There was also a nonsignificant difference found between both intervention groups for the change in vegetable intake (β=63.372, SE 36.667; χ^2^_1_=3.0, *P*=.08): a greater increase in vegetable intake from baseline (mean 120.137, SD 38.800 g/day) to posttreatment (mean 220.637, SD 38.800 g/day) was found for adults in the GPs’ intervention group than the increase from baseline (mean 152.502, SD 20.830 g/day) to posttreatment (mean 189.630, SD 20.830 g/day) in the researchers’ intervention group.

## Discussion

### Principal Findings

This study evaluated the effectiveness of the newly developed Web-based intervention MyPlan 1.0 on the fruit and vegetable intake of adults who visit general practice. The high percentage of adults in our sample who did not reach the guidelines for fruit and vegetable intake at baseline emphasizes the need for effective interventions. MyPlan 1.0 has the potential to increase self-reported fruit and vegetable intake in adults. Greater increases in self-reported fruit and vegetable intake were reported in both intervention groups (received MyPlan 1.0) compared to the control group (received usual care). In most (Flemish) general practices, health promotion involves the provision of flyers and leaflets with general information on dietary behavior [23]. Thus, the findings of our study provide superior effects for the use of self-regulation-based/tailored eHealth dietary interventions. Although MyPlan 1.0 was effective in increasing fruit and vegetable intake of adults, a large part of the participating adults did not reach health guidelines for fruit and vegetable intake after 1 month. This may be because MyPlan 1.0 did not instruct adults to immediately pursue health norms. Rather, adults were stimulated to set personal and attainable health goals (eg, eating one portion of fruit every day) and to reach the health norms over time. Therefore, more research is needed to evaluate the long-term effects of the intervention.

The review of Bhattarai et al [28] showed that interventions that promote healthy diet in primary care have the potential to improve fruit and vegetable intake over 12 months. However, studies included in this review that targeted fruit and vegetable intake made use of face-to-face contacts or motivational phone calls. To our knowledge, no other Web-based interventions based on self-regulation that target fruit and vegetable intake in adults have been evaluated in general practice yet.

In the review of Ball et al [29], it was concluded that effectiveness of dietary interventions implemented in primary care may depend on the theoretical underpinning and content of the intervention. A strength of our study is that MyPlan 1.0 is indeed theory based because it includes several aspects of self-regulation theory and incorporates several self-regulation behavior change techniques. However, further investigation about which (or combinations of which) behavior change techniques are effective is needed. The design of our study did not allow us to examine whether all components were effective and whether a particular combination is necessary. Future research should evaluate the individual impact of the different intervention components.

Ball et al [29] also indicated that intervention effectiveness may be influenced by the way of delivery in primary care. Because GPs have been put forward as a credible and reliable source for health promotion [21,22], it was suggested that GPs’ influence may also play an important role in intervention effectiveness of MyPlan 1.0. However, in our study, we did not find significant differences in effect between the GPs’ intervention group and the researchers’ intervention group. Yet, it has to be noted that due to low reach and high dropout, only a small group completed the intervention in the GPs’ intervention group at 1 month, resulting in restricted statistical power. This may explain why no significant differences in effects were found between the two intervention groups. Intention-to-treat analyses were also conducted in which missing values of mean fruit/vegetable intake at T2 were replaced by values reported at T0 (baseline) or T1 (after 1 week). These analyses showed similar results, but also showed a greater increase in vegetable intake in the GPs’ intervention group compared to the researchers’ intervention group, although this was not statistically significant. These results might indicate that implementing Web-based interventions in routine practice of primary care settings could lead to beneficial effects. However, this needs to be evaluated in further research. The marginally better outcomes in the GPs’ intervention group may also be the result of which adults were recruited by the GP (eg patients with more room for improvement). Our results indicated that GPs recruited a different sample of participants. The BMI of participants in the GPs’ intervention group was higher (mean 26.50, SD 5.11 kg/m^2^) compared to BMI of participants in the researchers’ intervention group (mean 25.99, SD 5.65 kg/m^2^), although the difference was not statistically significant. However, in this study, the aim was to use MyPlan 1.0 in the general population and not only for secondary prevention. Therefore, in both intervention conditions, researchers and GPs were instructed to recruit adults that were 18 years or older. The bias selection and the low reach (n=41) in the GP group may indicate that GPs had difficulties recruiting adults as they were instructed. In previous research, several obstacles were also reported by GPs to implement health promotion interventions, such as lack of training and skills, lack of time, difficulties addressing adults with no related complaints, other priorities in patient care, lack of resources, skepticism about efficacy, and GPs perceiving other health professionals as better suited [21,22,30-34]. We tried to overcome these obstacles by involving GPs from the beginning of the developmental process of MyPlan 1.0 (eg, through focus group interviews) [23], by offering different choice options to GPs to implement the intervention, and by providing minimal instructions to GPs. Still, implementing an intervention by GPs seems to be less feasible, which may indicate that GP involvement in recruitment for health promotion interventions is not recommended for future research. Because our results also showed strong effects in the researchers’ intervention group, delivery by others in general practice (eg, by a practice assistant) or in other (primary care) settings and contexts (eg, pharmacists, dieticians, work places) could also be considered and evaluated.

### Limitations

An unexpectedly high attrition rate was observed in the intervention group (71.8%, 199/277) and in the control group (59.1%, 88/149). Previous studies have also reported high dropout rates and low levels of sustained use of Internet-delivered interventions [17]. Therefore, suggestions of those studies were followed to prevent dropout by providing personal feedback, facilitating goal setting and self-monitoring of behavior, the use of periodic email reminders and incentives, and the provision of counselor support [17,35]. However, the dropout rate in the GPs’ intervention group was as high as in the researchers’ intervention group. This is in contrast with other studies that showed that the use of GP endorsement resulted in improved response to postal questionnaires in health care research [35,36]. Perhaps GPs in our study were not sufficiently involved in the behavior change process and did not motivate participants enough compared to in other studies in which GPs were involved more extensively. Another reason could be that filling in research questionnaires used for the study required too much time and effort. This may increase dropout rates for the research part, but does not necessarily mean drop out from the intervention.

Our dropout analyses indicated that men and participants with low education were more likely to drop out. This is in line with previous studies that identified participant characteristics related to sustained use. Participants who complete health interventions tend to be female, middle-aged, and higher educated [17,37-39]. So, the fact that more lower educated adults participated in our study can perhaps explain the higher dropout rates. This argues for a further evaluation of strategies to prevent dropout, especially in lower educated adults. Furthermore, we also found that participants who chose more than one health behavior were more likely to drop out. Letting participants choose multiple behaviors was included as a possibility to increase participants choices, based on the principles of the self-regulation perspective. Furthermore, previous studies have shown that interventions that target multiple behavioral changes simultaneously may have a greater impact than single-behavior interventions [36]. However, self-regulation capacity of adults’ has been shown to be limited and it might be difficult for adults to make multiple behavior changes at the same time [40]. This may explain higher dropout when choosing multiple behaviors.

A consequence of the high dropout and smaller sample size is that this may lead to nonsignificant results, so further research is needed to verify whether or not there truly is an effect.

A second limitation is the use of a quasi-experimental design, which may have resulted in nonequivalent groups. Therefore, increases in fruit and vegetable intake over time may not only be attributed to the intervention, but also to other differences in variables between the groups. Therefore, analyses were controlled for confounding variables (socioeconomic status, age, sex, reaching health norms). Moreover, no fidelity check procedures were conducted on how GPs motivated participants to participate in the study and how GPs discussed participants’ advice/action plans. Another limitation is that we did not track or measure website use and user experience and, therefore, have no information on the impact of website use on the intervention effects. Further, self-reported data can lead to reporting biases, although it is difficult and extremely costly to measure dietary behaviors objectively [41]. Therefore, the use of validated questionnaires was important.

Finally, the short study duration must be taken into account when interpreting the results. It may be that intervention effects decline after intervention completion [41,42]; however, it is also possible that intervention effects increase over time when using a self-regulation approach. To evaluate this, long-term follow-up measurements in future research are necessary.

### Conclusions

In conclusion, this study showed a greater increase in fruit and vegetable intake when the Web-based intervention MyPlan 1.0 was used compared to usual care of health promotion in general practice (ie, general information). Thus, the findings of this study add new evidence for the further evaluation and use of Web-based dietary interventions implemented through primary care. However, the short study duration and large dropout rate also implicate that more research is needed on the long-term effects of the intervention and that strategies to prevent dropout should be evaluated. The bias selection and low reach in the GP intervention group also showed that GP involvement in recruitment may not be recommended for future research. Finally, further investigation on which (or combinations of which) self-regulation behavior change techniques are effective is needed.

## Appendix

Multimedia Appendix 1 Flowchart for GPs.

## Abbreviations

BMI: body mass index
GP: general practitioner
IGLS: iterative generalized least squares
MCAR: missing completely at random
