# Supplementary figures and images for: Effect of the Web-Based Intervention MyPlan 1.0 on Self-Reported Fruit and Vegetable Intake in Adults Who Visit General Practice: A Quasi-Experimental Trial
Source: J Med Internet Res. 2016 Feb 29;18(2):e47. doi: 10.2196/jmir.5252 (PMC4791527; doi:10.2196/jmir.5252)

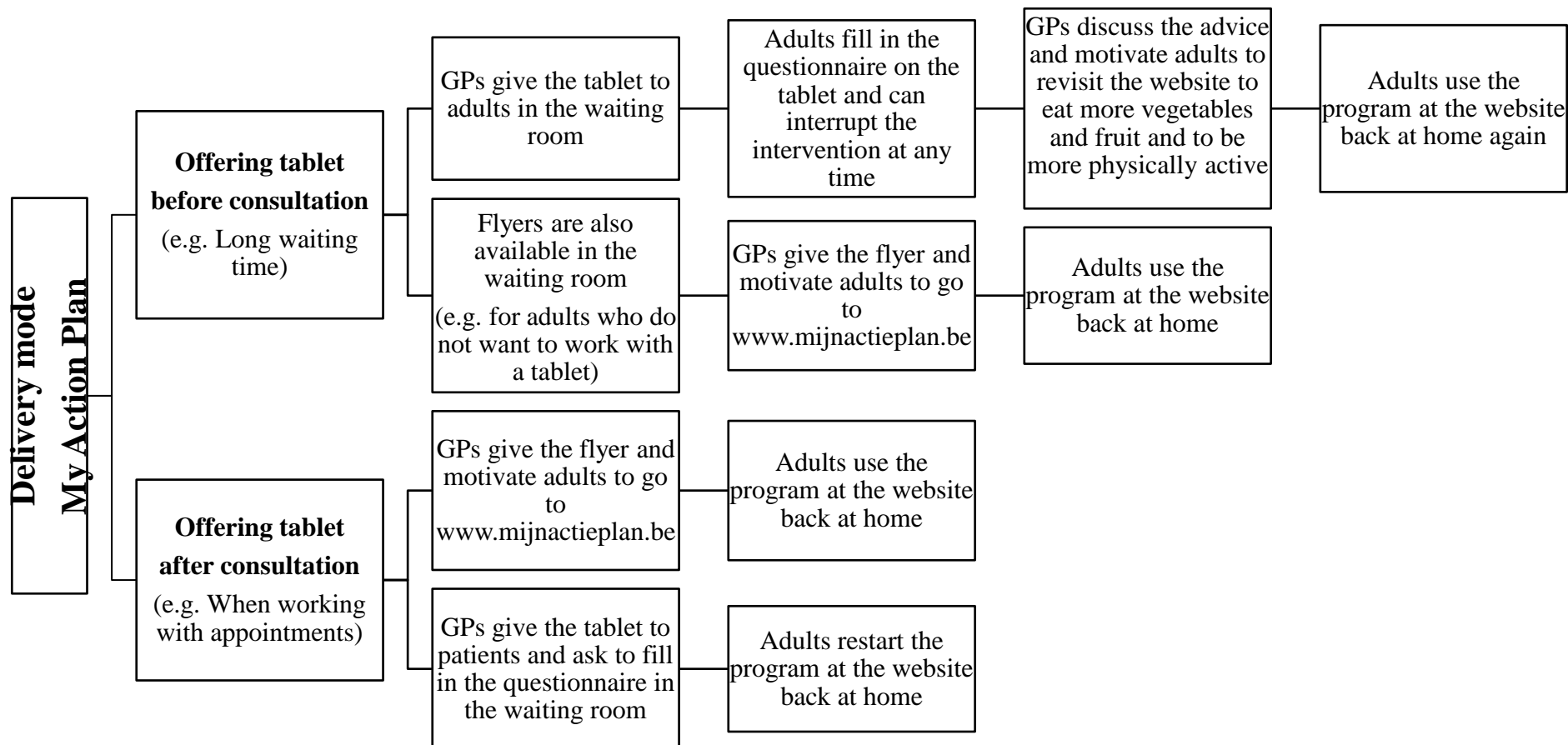

Supplement: Multimedia Appendix 1 [file jmir_v18i2e47_app1.pdf]
